# The Trickle-Down Effect of Authoritarian Leadership on Unethical Employee Behavior: A Cross-Level Moderated Mediation Model

**DOI:** 10.3389/fpsyg.2020.550082

**Published:** 2021-01-06

**Authors:** Jiang Rui, Lin Xin Qi

**Affiliations:** ^1^Business School, Hohai University, Nanjing, China; ^2^School of Labor and Human Resources, Renmin University of China, Beijing, China

**Keywords:** authoritarian leadership, unethical employee behavior, trickle-down effect, LMX, ethical climate

## Abstract

Authoritarian leadership is of great significance to eastern countries, including China. Meanwhile, unethical employee behavior also exists in all types of social organizations. The relationship between authoritarian leadership and unethical employee behavior is worth studying. Senior leaders (managers) often do not have a direct influence on employees except for through their immediate supervisors. The leadership style of senior leaders also influences the leadership style of their subordinates (employees’ direct supervisors). This paper studies how authoritarian manager leadership trickles down to unethical employee behavior through authoritarian supervisor leadership (through social learning theory and ASA theory) and discusses the moderating effect of leader member exchange (LMX) and an ethical climate. Through a questionnaire survey of 406 pairs of leaders, supervisors, and employees, the research results of the multilevel model show that (1) authoritarian supervisor leadership is positively related to unethical employee behavior, (2) authoritarian supervisor leadership mediates the relationship between authoritarian manager leadership and unethical employee behavior, (3) LMX positively moderates the relationship between authoritarian manager leadership and authoritarian supervisor leadership and moderates the mediating effect of authoritarian supervisor leadership, and (4), that an ethical climate negatively moderates the relationship between authoritarian supervisor leadership and unethical employee behavior and moderates the mediating effect of authoritarian supervisor leadership.

## Introduction

Previous research has shown that there are factors that influence the unethical behavior of employees, such as time pressure (Koh et al., 2018), the organizational climate (Zaal et al., 2019), and challenging performance goals (Welsh et al., 2019). Leadership is a key contextual factor and has been proved to play an important role in unethical behaviors in employees. However, existing research on the influence of unethical employee behavior mainly focuses on the leadership styles proposed in a western context, and relatively little is known about leadership styles in non-western contexts, such as the leadership style in the context of eastern countries, including China, where authoritarian leadership is more common. Authoritarian leadership is a leadership style rooted in traditional culture, which means that the leader has absolute authority over an individual in the management process and takes strict measures to monitor employees (Zheng and Huang, 2000). There is a clear difference between authoritarian leadership and the leadership style proposed in the western context, for example, servant leadership includes guiding, supporting democracy, and delegation (Chughtai, 2019). Transformational leadership includes morals, a vision to inspire, personalized care, and leadership charm (Parveen and Adeinat, 2019). Ethical leadership mainly refers to situations in which leaders require themselves and their subordinates to behave in an ethical way (Haar et al., 2019).

It is of great theoretical and practical significance to study the relationship between authoritarian leadership and unethical employee behavior. Existing studies on the consequences of authoritarian leadership mainly focus on voice behavior (Chou and Long, 2014), the creativity of employees, and employee performance, with research results that show the negative effects of authoritarian leadership. This paper extends this research on the impact of authoritarian leadership to unethical employee behavior. Past studies of unethical employee behavior usually adopt a perspective that mainly focuses on ethical leadership (Kalshoven et al., 2011; Joosten et al., 2014) and responsibility leadership (Graham et al., 2018), which are mainly leadership styles in western-oriented situations. This paper is among the first to study the positive influence of Chinese traditional authoritarian leadership on unethical employee behavior, which enriches theoretical research on unethical employee behavior in the Chinese context. In practice, on the one hand, authoritarian leadership is a representative dimension of paternalistic leadership, and its research is more helpful to eastern organizations, including China; on the other, unethical employee behaviors occur in various organizations, and it is meaningful to explore their roots, to reduce such behaviors. Therefore, further research on the relationship between authoritarian leadership and unethical employee behavior can help us find solutions to reduce unethical employee behavior. From a review of relevant literature, we found that there may be a relationship between the two behaviors.

The trickle-down effect refers to not giving poor vulnerable groups or poor areas preferential treatment in terms of economic development, but the priority groups or regions benefit the poorer classes or regions through consumption, employment, and other aspects that drive their development and prosperity. The trickle-down effect is an economic concept known as the pass-through effect. We believe that this effect also exists in manager leadership behavior and superior leadership behavior. The existing literature studies the influence of direct supervisor leadership behaviors on employees, including the authoritarian leadership of supervisors. However, the formation of an employee’s direct supervisor leadership style is likely to be influenced by the leadership style of senior leaders (managers); thus, it is necessary to study how supervisor leadership style is affected by the leadership style of senior leaders and how it ultimately affects the employee’s behavior. This study attempts to make some contributions in this respect.

**Figure 1:**
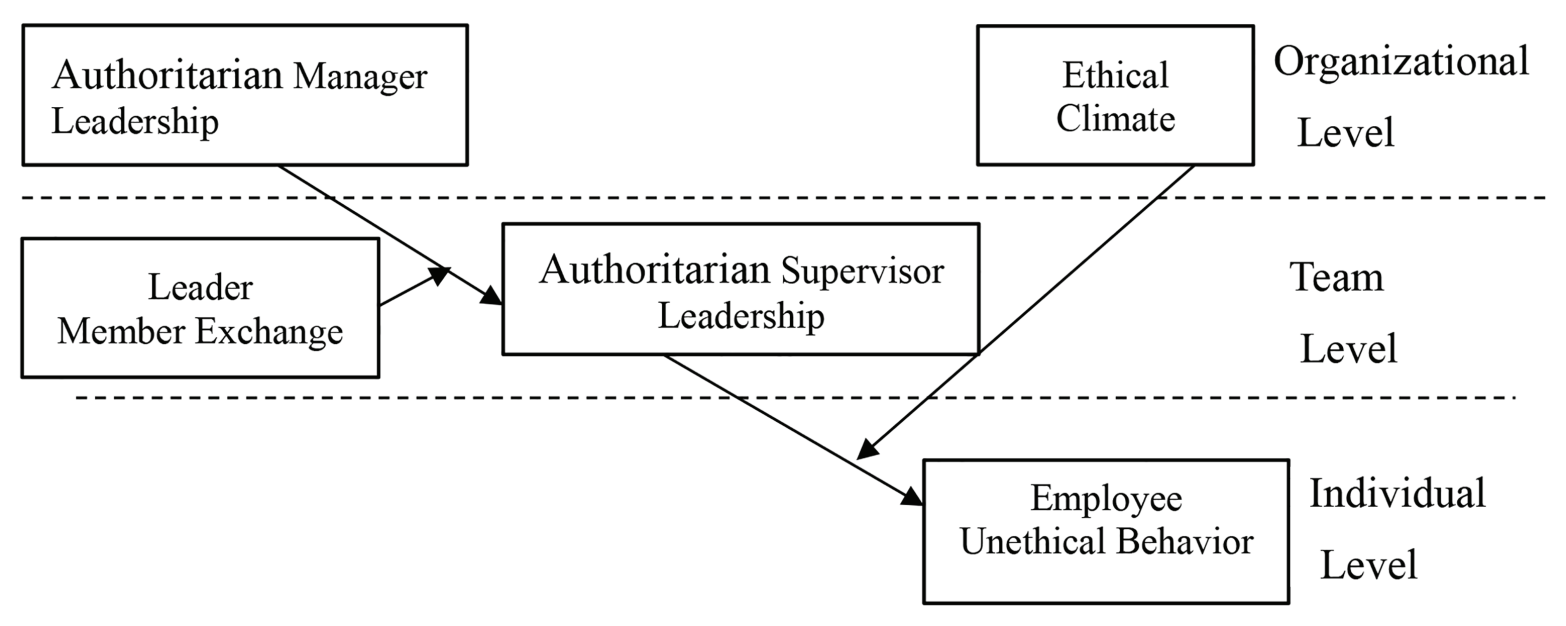
Theoretical model.

This paper discusses how authoritarian manager leadership can be transmitted to unethical behavior in employees through authoritarian supervisor leadership. On this basis, this paper studies the situational factors that affect the trickle-down effect and the moderating effect of leader member exchange (LMX), and argues that authoritarian manager leadership is more likely to affect authoritarian supervisor leadership under high LMX than under low LMX because, under high LMX, supervisors are more likely to imitate the leadership style of their managers. This has not been addressed in previous studies. Finally, we believe that the influence of authoritarian leadership on unethical employee behavior is moderated by the ethical climate of the wider organization, which can help enhance the influence of authoritarian leadership on unethical employee behavior, for under positive ethical climate employees are influenced by climate to reduce unethical behaviors (Figure 1).

## Theory and Hypotheses

### Authoritarian Supervisor Leadership and Unethical Employee Behavior

Unethical employee behavior is defined as behavior that violates the moral code widely accepted by people (Trevino et al., 2014) and has strong concealment and dangers, breeds corruption easily, leads to inefficiencies, and increases the losses of an organization (Pierce and Aguinis, 2015). Authoritarian leadership emphasizes the absolute personal authority of the leader and the absolute obedience of the subordinate, which reflects the strong hierarchical relationship between superior and subordinate (Zheng and Huang, 2000). After years of research on authoritarian leadership, Zheng and Fan (2000) concluded that authoritarian leadership includes four dimensions, namely, “authoritarian style,” “image grooming,” “derogating subordinates’ ability,” and “teaching.” “Authoritarian style” means that the superior centralizes power, closely monitors subordinates, and does not communicate with the subordinate. “Image grooming” refers to how the leader maintains their image in front of the staff, giving people a sense of confidence. “Derogating subordinates’ ability” means that the superior will belittle the ability of the subordinate, that they do not accept the subordinate’s suggestions, and avoid praising the employee. “Teaching” behavior refers to a scenario where the superior fosters a high performance standard to the subordinate and gives a direct reprimand to subordinates whose performance is not ideal (Zhou and Liao, 2012).

From the perspective of diversion attack, Tepper et al. (2008) found that employees who had experienced unfair treatment of workplace oppression and breach of psychological contract were more likely to transfer unethical behavior to other individuals or the wider environment, which could help them repair their damaged emotions. In research on diversion attacks, unethical behavior in employees was regarded as a negative emotion processing strategy to digest workplace stressors. When supervisors use authoritarian leadership, it will bring negative psychological experience and pressure to employees, and employees will transfer such negative psychological experiences and pressures to unethical behavior. Meanwhile, as an extension of social cognition theory, the concept of moral liberation proposed by Bandura (1986) explains how individuals can influence their thoughts and behaviors through a self-regulation process and conduct behaviors that violate basic social ethics. The essence of moral liberation is a series of cognitive mechanisms that invalidate the process of moral self-adjustment, including moral defense, favorable contrast, euphemistic labeling, buck-passing, responsibility diffusion, neglect, or distortion of the result, dehumanization, and attribution of responsibility (Bandura, 1986). According to the theory of moral liberation, employees are more likely to exhibit unethical behavior when they reduce their guilt by attributing it to a supervisors’ unreasonable authoritarian leadership. Therefore, we propose the following:

*Hypothesis* 1: Authoritarian supervisor leadership is positively related to unethical employee behavior.

### The Mediating Role of Authoritarian Supervisor Leadership

We use social learning theory to explain the transmission effects of leadership. According to social learning theory, when a model exists, individuals tend to emulate the model in their behavior (Simon and Mark, 2012). In the workplace, leaders have absolute authority to dominate resources, evaluate employee performance and make decisions; therefore, subordinates may emulate this behavior to obtain more resources (Simon and Mark, 2012; Miao et al., 2013). This can for example occur between managers and supervisors of employees (Bennett and Robinson, 2000). Mayer (2009) found that ethical manager leadership is positively related to the ethical leadership of supervisors, i.e., ethical leadership can pass from managers to supervisors. We can also predict that the trickle-down effect exists in authoritarian leadership. When the supervisors of employees see managers using authoritarian leadership to manage their subordinates, they may feel that this is a sound and effective leadership style. Thus, they will emulate the behavior of their managers consciously and unconsciously, especially in high power distance countries (e.g., China), where respect and obeying orders are valued.

We can also use the attraction-selection attrition (ASA) model to explain the relationship between authoritarian manager leadership and authoritarian supervisor leadership. The ASA model suggests that employees who have similar personalities, attitudes, and values to those of an organization are more likely to be attracted to and selected in that organization (Schneider, 1987), indicating that managers are more likely to select subordinates (supervisors) similar to themselves (Ning et al., 2012). Thus, it follows that managers with an authoritarian leadership style are more likely to choose people with authoritarian leadership characteristics as subordinates and to give them more opportunities for advancement. To obtain more trust and promotion opportunities, the supervisors of employees will try their best to maintain a leadership style that is in accord with that of their managers.

Authoritarian leadership consists of four components: “authoritarian style,” “image grooming,” “derogating subordinates’ ability,” and “teaching.” From the perspective of diversion attack (Tepper et al., 2008), when supervisors use authoritarian leadership, it will bring negative psychological experiences and pressure to employees, and employees will react by transferring these negative psychological experiences and pressures into unethical behaviors. According to moral liberation theory (Bandura, 1986), employees are more likely to exhibit unethical behavior and reduce their guilt by attributing it to the unreasonable authoritarian leadership of supervisors. Therefore, we propose the following:

*Hypothesis* 2: Authoritarian supervisor leadership mediates the relationship between authoritarian manager leadership and unethical employee behavior.

### The Moderating Role of LMX (Manager-Supervisor Exchange Quality)

Leadership member exchange describes the quality of the exchange relationship between leaders and subordinates in a formal organization (Graen and Uhl-Bien, 1995). Due to time pressures and resource constraints, supervisors often treat subordinates differently. Leaders will establish high-quality exchange relationships of mutual trust and respect with some subordinates who are called “insiders.” In contrast, the relationship between the leader and another group of subordinates is limited to the job description, and these subordinates are called “outsiders” (Graen and Uhl-Bien, 1995). In high-quality LMX relationships, leaders give members more resources and support, and members gain more freedom, more decision-making rights, and organizational support (Victor and Cullen, 1988; Farh et al., 2007; Thau et al., 2015). In return, subordinates tend to following the leader. Supervisors try their best to demonstrate a leadership style similar to that of the leader when managing employees, to obtain recognition and appreciation from their managers. In contrast, in low-quality LMX relationships, supervisors are not willing to show similar behaviors to gain recognition from managers. Therefore when managers use authoritarian leadership in high-quality LMX leadership, supervisors are more likely to show authoritarian leadership to get recognition and appreciation.

Under different LMX qualities, the subordinate has a different desire to learn the behavior of the superior. When the manager-supervisor exchange quality is high, the supervisor is more willing to learn the authoritarian leadership style of the manager. In contrast, when manager-supervisor exchange quality is low, the supervisor is less willing to learn the authoritarian leadership style of the manager. Therefore, we propose the following:

*Hypothesis* 3: LMX (manager-supervisor exchange quality) positively moderates the positive relationship between authoritarian manager leadership and authoritarian supervisor leadership, that is, the higher the quality of the leadership exchange, the stronger the positive correlation between authoritarian manager leadership behavior and authoritarian supervisor leadership behavior.

Authoritarian manager leadership affects unethical employee behavior through authoritarian supervisor leadership. We believe that LMX moderates the mediating effect of authoritarian supervisor leadership, as it has a moderated mediating effect. In a high-quality LMX relationship, to gain recognition and more resources, supervisors who are “insiders” will imitate the authoritarian leadership of their managers. Meanwhile, managers are more likely to select supervisors who have a similar leadership style. Therefore, authoritarian manager leadership is more likely to trickle down to supervisors; thus, the unethical behavior of employees decreases correspondingly when supervisors use authoritarian leadership. In contrast, in a low-quality LMX relationship, supervisors are not willing to show authoritarian leadership similar to that of their managers, which may increase unethical employee behavior. Therefore, we propose the following:

*Hypothesis* 4: LMX (manager-supervisor exchange quality) moderates the mediating effect of authoritarian supervisor leadership on the relationship between authoritarian manager leadership and unethical employee behavior. That is, the higher the quality of LMX, the stronger the indirect relationship between authoritarian manager leadership and unethical employee behavior through authoritarian supervisor leadership.

### The Moderating Role of an Ethical Climate

An organizational ethical climate is the wider perception of ethical standards by employees within an organization and the ethics of managers, which involves punishment for violations of the system (Schwepker, 2001). This common perception is formed by the members of an organization as to what ethical behaviors are and how to address them. The ethical climate in different organizations and departments can be strong or weak (Victor and Cullen, 1988). There are five types of organizational ethics: self-interested, caring, rules-based, legal and professional, and independent judgment (Victor and Cullen, 1988). According to social exchange theory, LMX based on respect and trust often produces long-term reciprocal relationships based on emotion. In the context of highly caring organizational ethics, because of the love and care of the organization or its members, employees put collective interests first when making choices out of gratitude and avoid unethical behavior that damages the collective interest. In an ethical climate with a high emphasis on rules and regulations, the organization requires employees to follow the rules and regulations and industry norms, i.e., the conduct of the organization and its members is strictly governed by such rules and regulations. Under such strong rules and regulations, employees also minimize unethical behavior that violates organizational rules and regulations to avoid punishment. In highly legal and code-of-conduct-oriented organizational ethical climates, social and industrial laws and codes of practice impose strong constraints on employees, meaning that they avoid unethical behavior that violates laws and codes of conduct. Therefore, a positive organizational ethical climate can help authoritarian leadership reduce unethical behaviors in employees. In a negative organizational ethical climate, employees will ignore the collective interest, rules, regulations, social laws, and professional codes. When supervisors use authoritarian leadership, unethical employee behavior will increase, and the relationship between authoritarian supervisor leadership and unethical employee behavior will become stronger. Therefore, we propose the following:

*Hypothesis* 5: An ethical climate negatively moderates the positive relationship between authoritarian supervisor leadership and unethical employee behavior. That is, the higher the level of ethical climate, the weaker the positive correlation between authoritarian leadership behavior and supervisors, and unethical behavior in employees.

Furthermore, we believe that the ethical climate moderates the mediating effect of authoritarian supervisor leadership. It is a moderated mediating effect. In a positive ethical climate, because employees consider the collective interest and abide by the rules and regulations of the organization, social laws, and professional ethics, the unethical behavior of employees will be reduced under authoritarian supervisor leadership. In contrast, in a negative ethical climate, employees think more about their interests and ignore the rules and regulations of the organization, social laws, and professional ethics. Thus, when personal interest conflicts with organizational interests, employees will choose personal interests, violating rules and regulations of the organization, social laws, and professional ethics. The results of research also indicates that an ethical climate moderates the relationship between leadership and organizational citizenship behavior. Thus, we can deduce that the ethical climate moderates the relationship between authoritarian leadership and unethical employee behavior as opposed to organizational citizenship behavior. Therefore, we propose the following:

*Hypothesis* 6: An ethical climate moderates the mediating effect of authoritarian supervisor leadership on the relationship between authoritarian manager leadership and unethical employee behavior. The higher the ethical climate, the weaker the indirect relationship between authoritarian manager leadership and unethical employee behavior through authoritarian supervisor leadership.

## Sample and Measures

### Sample and Procedure

We chose 24 companies in China as samples, including manufacturing, real estate, food processing, and finance, etc. The companies were from Jiangsu, Shanghai, Beijing, Zhejiang, Chongqing, and Wuhan. A total of 95 working teams were chosen as samples, and the respondents were composed of team leaders and other team members. Every team was composed of at least one leader and other members to ensure that sufficient samples were collected. The scale of authoritarian supervisor leadership and the ethical climate was completed by employees, and the scale of authoritarian manager leadership, LMX, and unethical employee behavior was completed by supervisors. From July to October 2016, the author issued questionnaires to supervisors and employees in 24 companies. Before the investigation, we obtained permission and authorization from the person in charge of the company. In addition, with the cooperation of human resources departments, the questionnaire was immediately distributed to respondents. When filling in the questionnaire, the investigators explained the confidentiality of the investigation to the respondents and gave them 100RMB per person. After the questionnaires were completed, the respondents sealed the questionnaires in envelopes and gave them to the researchers. Although 536 questionnaires were distributed, 435 were completed by respondents. Finally, 406 sets of valid questionnaires were obtained from 90 effective teams of 24 enterprises, of which 406 questionnaires were valid. The response rate was 75.7%.

The proportion of male and female participants were almost the same, with 52.1% being male. In terms of their age, most respondents were either middle-aged or young, i.e., 4.5% were under 20 years of age, 56.9% were aged between 20 and 30, 31.6% were aged between 30 and 40, 5.3% were aged between 40 and 50, and 2.7% were above 50 years. In terms of marriage, the proportion of married individuals in the sample was 42.5%. In terms of education level, 9.5% had completed high school, 25.6% were at the post-secondary level, 49.5% were at an undergraduate level, and 15.4% were at Master’s level or above.

### Measures

By searching relevant literature, we selected mature scales in which LMX, unethical employee behavior, and ethical climate are English scales. To ensure the validity of the scales, we used the “translation and back translation” procedure. A five-point scale was used for all the questionnaires, from “1” strongly disagree to “5” strongly agree.

### Authoritarian Leadership

This paper chose an authoritarian leadership dimension that encompassed three dimensions of paternalistic leadership developed by Zheng and Fan (2000), including 13 items. An item example is “In front of us, the leader is very dignified in appearance.” In the study, the coefficient of the internal consistency of the scale of authoritarian manager leadership (Cronbach’s Alpha) is 0.796, and the coefficient of the internal consistency of the scale of authoritarian supervisor leadership (Cronbach’s Alpha) was 0.784.

### Unethical Employee Behavior

This study adopted the scale developed by Newstrom and Ruch (1975), including 17 items. Two example items are “Take credit for the accomplishments to others,” “Shirk responsibilities to colleagues.” This scale is widely used within and outside of China and proved reliable. This scale was filled-in by supervisors. In the study, the coefficient of the internal consistency of the scale (Cronbach’s Alpha) was 0.747.

### Leader Member Exchange

This study adopts the scale developed by Graen and Uhl-Bien (1995), including 7 items. Two example items are “The leader knows about the problems and demands of my job,” “I get along well with the leader at work.” This scale is used frequently in China and has proven to be reliable in other studies. This scale is filled in by supervisors. In the study, the coefficient of the internal consistency of the scale (Cronbach’s Alpha) was 0.725.

### Ethical Climate

This study adopts the scale developed by Schwepker (2001), including 13 items. An example item is “Our company has a formal code of ethics.” This scale is used in empirical research in China and has proven reliable (Ma and Du, 2014). This scale is filled in by employees. In the study, the coefficient of the internal consistency of the scale (Cronbach’s Alpha) was 0.834.

### Control Variables

Past literature indicates that employee gender, age, marriage, educational level, and time on the team are related to unethical employee behavior to a certain extent. Therefore, this research chose these variables as control variables.

### Analytical Approach

We used Mplus 8.0 to conduct multilevel path analysis, using a nested research design. The variable at level 1 is unethical employee behavior, variables at level 2 are authoritarian supervisor leadership and LMX, while authoritarian manager leadership and ethical climate are at level 2. We first undertook a data aggregation test, and second, performed confirmatory factor analysis, third, we used a common method bias test, and finally, we tested the hypotheses.

## Results

### Data Aggregation Test

Because the scale pertaining to authoritarian supervisor leadership and the ethical climate was completed by employees and the authoritarian manager leadership and LMX scales were completed by supervisors, it is necessary to test their internal consistency (*Rwg*) and internal correlation (*ICC*), to aggregate them at the team or organizational level for testing. The calculated *Rwg* and *ICC* values are shown in Table 1, in which we found that the *Rwg* values met the standard of >0.7; also, the *ICC* (1) are higher than 0.12 and *ICC* (2), satisfying the criteria of being above 0.7. Therefore, it is possible to aggregate these values at the team or organizational level.

**Table 1:** Data aggregation analysis results.

Variable (team/organizational level)	*Rwg*	*ICC (1)*	*ICC (2)*
Authoritarian supervisor leadership	0.812	0.236	0.906
Ethical climate	0.854	0.175	0.879
Manager supervisor leadership	0.807	0.201	0.918

### Confirmatory Factor Analysis

Based on the above findings, this study adopted confirmatory factor analysis (CFA) to test the structural validity of major variables. From Table 2, we can see that the five-factor model fits well (*χ*^2^ (271) = 860.211, *χ*^2^/*df* = 3.174, RMSEA = 0.053, CFI = 0.959), and is significantly better than other factor models. From *χ*^2^ and AIC, we can see that the five-factor model is also significantly better than the other alternative models, which indicates that variable discriminant validity is verified.

**Table 2:** Confirmatory factor analysis.

Model	*χ*^2^	*df*	*χ*^2^/*df*	AIC	NNFI	CFI	RMR	RMSEA
Five factors: AML, ASL, EUB, LMX, EC	860.211	271	3.174	88.306	0.942	0.959	0.033	0.053
Four factors: AML + ASL, EUB, LMX, EC	946.334	275	3.441	191.541	0.738	0.814	0.056	0.101
Four factors: AML + LMX, ASL, EC, EUB	1215.205	275	4.419	237.348	0.677	0.752	0.065	0.133
Four factors: AML, ASL + EC, LMX, EUB	1563.157	275	5.684	259.825	0.620	0.708	0.069	0.145
Tree factors: AML + ASL + LMX, EC, EUB	1834.496	278	6.599	289.105	0.582	0.638	0.074	0.152
Tree factors: AML, ASL + EC + EUB, LMX	2219.160	278	7.983	325.120	0.533	0.587	0.079	0.162
Two factors: AML + ASL + LMX + EC, EUB	2503.174	280	8.940	378.304	0.491	0.502	0.085	0.170
Single fators: AML + ASL + LMX + EC + EUB	3101.514	281	11.037	412.113	0.454	0.448	0.090	0.176

*N*(member) = 406; AML, authoritarian manager leadership; BSL, authoritarian supervisor leadership; LMX, leader member exchange; EC, ethical climate; UEB, unethical employee behavior; +, represents the combination of two factors into a variable.

### Descriptive Statistics

Table 3 shows that authoritarian manager leadership is significantly related to authoritarian supervisor leadership (*r* = 0.288, *p* < 0.01), ethical climate (*r* = −0.253, *p* < 0.05), and unethical employee behavior (*r* = 0.135, *p* < 0.01). Authoritarian supervisor leadership is significantly related to LMX (*r* = 0.231, *p* < 0.05), ethical climate (*r* = −0.102, *p* < 0.05) and unethical employee behavior (*r* = 0.185, *p* < 0.01). The ethical climate is significantly related to unethical employee behavior (*r* = −0.434, *p* < 0.01). It is thus clear that these variables are significantly related to each other, which lays a foundation for further hierarchical regression analysis.

**Table 3:** Means, SD, and correlations between study variables.

Variable	*M*	*SD*	1	2	3	4	5	6	7	8	9	10
1.Gender	0.517	0.523	—									
2.Age	2.195	0.671	0.255^**^	—								
3.Education	2.437	0.852	0.192^**^	0.253^**^	—							
4.Marital status	0.410	0.460	0.231^**^	0.640^**^	0.106^*^	—						
5.Team time	2.253	1.388	0.069	0.454^**^	−0.059	0.345^**^	—					
6.EUB	2.302	0.459	0.065	−0.085	−0.134^*^	−0.125^*^	−0.246	—				
7.ASL	3.476	0.499	0.076	−0.098	−0.181	0.045	−0.072	0.185^**^	—			
8.LMX	3.478	0.487	0.037	0.025	0.013	0.037	0.085	0.093^*^	0.231^*^	—		
9.AML	3.596	0.438	0.043	−0.071	−0.162	0.034	−0.026	0.135^**^	0.288^**^	0.109	—	
10.EC	3.156	0.753	0.078	0.018	0.182	0.064	0.131	−0.434^**^	−0.102^*^	0.071	−0.253^*^	—

*N*(Member) = 406; AML, authoritarian manager leadership; ASL, authoritarian supervisor leadership; LMX, leader member exchange; EC, ethical climate; UEB, unethical employee behavior.

* *p* < 0.05;

** *p* < 0.01.

### Common Method Bias Test

In this study, authoritarian manager leadership, LMX, and unethical employee behavior were evaluated by supervisors, while the authoritarian supervisor leadership and ethical climate were evaluated by employees. Since the questionnaire survey method was mainly adopted, there may also be a common method bias. To test the influence of common method bias, we first used the Harman single-factor test to examine the level of common method bias in this study, such that all the items in the questionnaire are analyzed by factor analysis without rotation. The variation amount of the first principal component explanation is 22.206%, which does not account for half of the total variation explanation amount (68.14%), indicating that the influence of common method bias is not obvious in this study. Considering that the Harman single-factor test is only a relatively rough method, this study also used the controlled, untested, single method latent variable method to test the common method bias that exists in the measurement. The effect of common method bias is included in the aforementioned five-factor model as a latent variable, allowing all the measurement items of the research variables to be loaded on the latent variable of this method and testing the common method bias effect by comparing the fitting degree difference between the two models. The results show that the fitting indexes of the model, including the common method bias latent variables, are also good (*χ*^2^/*df =* 3.261, RMSEA = 0.065, NNFI = 0.925, CFI = 0.976). However, comparing the *df* and chi-square values of the two models, it is found that the *df* of the original five-factor model decreases by 5, while the chi-square value of the chi-square model increases by 17.197, which is less than the critical value of 24.700 when the chi-square value was 0.010. Compared with the original five-factor model, the fitting degree of the model incorporating the latent variables of common method bias is not significantly improved, which also indicates that the common method bias in the measurement in this study is to some extent not serious.

### Hypothesis Testing

In this study, we used Mplus 8.0 for multilevel path analysis and Monte Carlo simulation to test our hypothesis. In Table 4, we can see from model 3 that authoritarian manager leadership is positively correlated with unethical behaviors in employees (*γ* = 0.266, *p* < 0.05, 95% CI [0.02, 0.14]). This supports Hypothesis 1, as outlined in this paper. In model 4, there is a significant positive correlation between authoritarian supervisor leadership and employees’ unethical behaviors (*γ* = 0.389, *p* < 0.01, 95% CI [0.08, 0.21]), while the influence coefficient of authoritarian leadership on the unethical behaviors of employees changed from original γ = 0.266 (*p* < 0.05, 95% CI [0.3, 0.13]) to current *γ* = 0.247 (*p* < 0.05, 95% CI [0.02, 0.20]), which shows that authoritarian supervisor leadership plays a mediating role between the authoritarian manager leadership and unethical behavior in employees.

**Table 4:** Non-normalized coefficients of multilevel path analysis.

Variable	Authoritarian supervisor leadership				Unethical employee behavior					
	Model 1		Model 2		Model 3		Model 4		Model 5	
	*b*	*SE*	*b*	*SE*	*b*	*SE*	*b*	*SE*	*b*	*SE*
Authoritarian supervisor leadership							0.389^**^	0.032	0.386^**^	0.027
Leader member exchange			0.090^*^	0.045						
Authoritarian manager leadership × Leader member exchange			0.088^*^	0.062						
Organizational level										
Authoritarian manager leadership	0.251^**^	0.086	0.129^**^	0.063	0.266^*^	0.103	0.247^*^	0.019		
Ethical climate									−0.478^**^	0.105
Authoritarian supervisor leadership × Ethical climate									−0.157^*^	0.084
*τ*00	0.047		0.056		0.015		0.074		0.126	
*σ*^2^	0.107		0.211		0.124		0.197		0.218	
*R*^2^level-1	—		0.059		—		—		—	
*R*^2^level-2	0.275		0.298		0.573		0.586		0.601	

Individual *n* = 406, team level *n* = 95, organizational level *n* = 24.

* *p* < 0.05;

** *p* < 0.01.

We used Monte Carlo simulation to test the mediating effect of authoritarian supervisor leadership. The indirect effect of authoritarian manager leadership on employees’ unethical behaviors through authoritarian supervisor leadership is 0.189 [95% CI (0.05, 0.18)], and hypothesis 2 of this paper is tested. According to model 2, leader member exchange is related to authoritarian supervisor leadership significantly (*γ* = 0,090, *p* < 0.05, 95% CI [0.09, 0.27]), and the cross-level interaction between authoritarian manager leadership and leader member exchange have a significant influence on authoritarian supervisor leadership (*γ* = 0.088, *p* < 0.05, 95% CI [−0.16, −0.07]). The *R*^2^level-2 changed from 0.275 to 0.298, indicating that leader member exchange has a significant cross-level positive moderating effect between authoritarian manager leadership and authoritarian supervisor leadership, and hypothesis 3 in this paper is tested. According to model 5, an ethical climate is related to unethical employee behavior significantly (*γ* = −0.478, *p* < 0.01, 95% CI [−0.25, −0.10]), and the cross-level interaction between authoritarian supervisor leadership and ethical climate have a significant effect on employees’ unethical behavior (*γ* = −0.157, *p* < 0.05, 95% CI [0.06, 0.23]). Meanwhile, *R*^2^level-2 changes from 0.586 to 0.601, indicating that the ethical climate has a significant cross-level negative moderating effect between authoritarian supervisor leadership and employees’ unethical behavior, which tests Hypothesis 5 of this paper.

**Figure 2:**
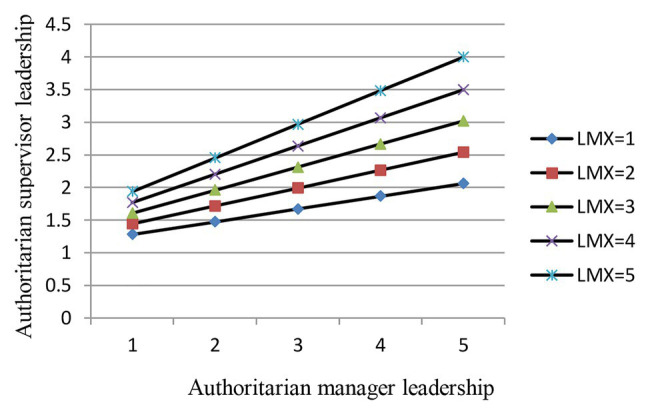
Moderating effect of LMX on the relationship between authoritarian manager leadership (AML) and ASL.

Figure 2 shows a graph of the relationship between authoritarian manager leadership and authoritarian supervisor leadership under both high and low LMX. From Figure 2, we can see that the positive relationship between authoritarian manager leadership and authoritarian supervisor leadership is stronger under high LMX than under low LMX. Therefore, hypothesis 3 is further verified.

**Figure 3:**
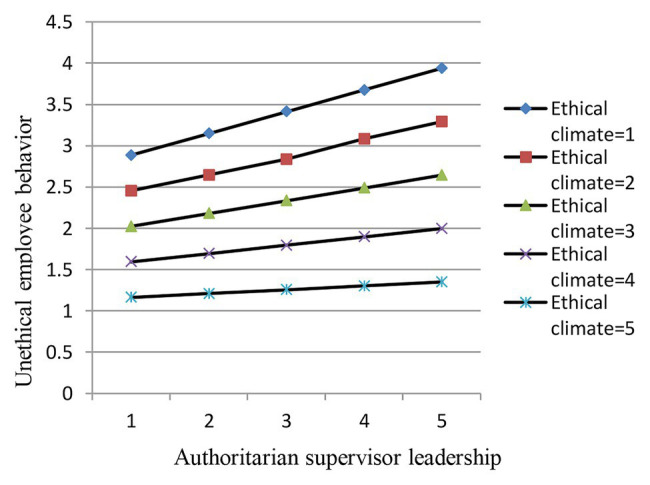
Moderating effect of ethical climate on the relationship between ASL and EUB.

Figure 3 shows a graph of the relationship between authoritarian supervisor leadership and unethical employee behavior under high and low ethical climates. Figure 3 indicates that the positive relationship between authoritarian supervisor leadership and unethical employee behavior is weaker under a high ethical climate compared to a lower ethical climate, further verifying hypothesis 5.

Next, we adopted the method suggested by Preacher et al. (2007) to test hypothesis 4, i.e., the moderating effect of LMX on the mediating effect of authoritarian supervisor leadership. From Table 5, we can see that under high LMX (one standard deviation above the mean), the mediating effect of authoritarian supervisor leadership on the relationship between authoritarian manager leadership and unethical employee behavior is 0.051 (SE = 0.015, *p* < 0.01), and under low LMX (one standard deviation below the mean), the mediating effect of authoritarian supervisor leadership is 0.038 (SE = 0.011, *p* < 0.01). The former is significantly lower than the latter, and the difference between high and low values is also significant (indirect effect = 0.013, SE = 0.014, *p* < 0.05). Meanwhile, the 95% CI values are not all zero under high or low LMX. Therefore, hypothesis 4 is supported.

**Table 5:** Moderating effect of leader member exchange (LMX) on the mediating effect of authoritarian supervisor leadership (ASL).

LMX	Mediating effect	*SE*	95% CI	
			Upper limit	Lower limit
High LMX	0.051^**^	0.015	0.027	0.096
Low LMX	0.038^**^	0.011	0.015	0.057
Difference between low and high	0.013^*^	0.014	0.008	0.052

*N*(member) = 406.

* *p* < 0.05;

** *p* < 0.01.

Finally, we again adopted the method suggested by Preacher et al. (2007) to test hypothesis 6, i.e., the moderating effect of ethical climate on the mediating effect of authoritarian supervisor leadership. From Table 6, we can see that in a highly ethical climate (one standard deviation above the mean), the mediating effect of authoritarian supervisor leadership on the relationship between authoritarian manager leadership and unethical employee behavior is 0.025 (SE = 0.016, *p* < 0.01), and in a low ethical climate (one standard deviation below the mean), the mediating effect of authoritarian supervisor leadership is 0.040 (SE = 0.013, *p* < 0.01). The former is significantly lower than the latter, and the difference between high and low values is also significant (indirect effect = −0.015, SE = 0.015, *p* < 0.05). Meanwhile, the 95% CI values are all not zero under high or low ethical climates. Therefore, hypothesis 6 is supported.

**Table 6:** Moderating effect of ethical climate on the mediating effect of ASL.

LMX	Mediating effect	*SE*	95% CI	
			Upper limit	Lower limit
High ethical climate	0.025^**^	0.016	0.024	0.090
Low ethical climate	0.040^**^	0.013	0.014	0.051
Difference between low and high	−0.015^*^	0.015	0.011	0.052

*N*(member) = 406.

* *p* < 0.05;

** *p* < 0.01.

## Supplementary Analysis

The volume and strength of the evidence presented by Gottfredson and Aguinis (2016) suggest that LMX is commonly a mediator. Therefore, we test whether LMX plays a mediating role in the relationship between authoritarian manager leadership and authoritarian supervisor leadership. As shown in Table 7, in model 1 authoritarian manager leadership is positively related to LMX significantly (*γ* = 0.173, *p* < 0.05, 95% CI [0.14, 0.32]), but in model 3, LMX is not related to authoritarian supervisor leadership (*γ* = −0.049, *p* > 0.05, 95% CI [−0.18, 0.05]). Therefore, LMX is not the mediator between authoritarian manager leadership and authoritarian supervisor leadership. This means LMX is a more suitable moderator.

**Table 7:** Mediating effect of LMX.

Variable	LMX	Authoritarian supervisor leadership				
	Model 1		Model 2		Model 3	
	*b*	*SE*	*b*	*SE*	*b*	*SE*
Authoritarian manager leadership	0.173^*^	0.120	0.251^**^	0.086	0.132^**^	0.185
LMX					−0.049	0.097
*τ*00	0.026		0.047		0.083	
*σ*^2^	0.091		0.107		0.139	
*R*^2^level-1	—		—		—	
*R*^2^level-2	0.155		0.275		0.318	

*N*(member) = 406.

* *p* < 0.05;

** *p* < 0.01.

## Conclusion and Discussion

Through empirical analysis, this study draws several conclusions: (1), authoritarian leadership is positively related to unethical employee behavior. This conclusion is consistent with the research results of other scholars. For example, studies by Li and Tian (2014) and Tian and Huang (2014) show that authoritarian leadership is an important factor that affects the psychology and behavior of employees. Combined with this finding, we believe that negative leadership styles tend to generate dissatisfaction and revenge in employees and ultimately lead to negative results. This result enriches research on the concepts of authoritarian leadership and unethical employee behavior. In contrast to previous research, this research focuses on leadership style in the Chinese context and unethical behaviors that frequently occur in organizations. The conclusions drawn from this research on the relationship between the two can be used for reference in both theory and practice to improve the leadership style of managers in Chinese organizations and reduce unethical employee behavior.

Our second conclusion is that (2) authoritarian supervisor leadership mediates the relationship between authoritarian manager leadership and unethical employee behavior. The results of this study tell us that the authoritarian leadership behaviors of supervisors in many situations can be traced back to the authoritarian leadership behaviors of managers. Meanwhile, the authoritarian leadership behaviors of managers do not directly affect the employees but rather influence the behaviors of employees through the leadership style of their supervisors. This research helps us to better understand the root causes of unethical employee behavior and the indirect process through which leadership influences employee behaviors.

The third conclusion is that (3) LMX moderates the relationship between authoritarian manager leadership and authoritarian supervisor leadership and the mediating effect of authoritarian supervisor leadership. LMX is sometimes used as a moderating variable to regulate the psychological or behavioral relationship between supervisors and subordinates. For example, studies by Lindsey et al. (2016), Newman et al. (2017), and Seo et al. (2018), used LMX as a moderating variable. This outcome indicates that managers must establish a high-quality LMX with supervisors to strengthen their influence.

Our fourth and final main conclusion (4) is that the ethical climate moderates the relationship between authoritarian supervisor leadership and unethical employee behavior and the mediating effect of authoritarian supervisor leadership. This result is consistent with the research conclusions of Shin (2012) and May et al. (2014), who also believe that regardless of the leadership approach, the ethical climate can be conducive to positive effects, especially on ethical behaviors. Creating a good organizational climate can help guide positive behaviors in employees, curbing negative behaviors.

### Theoretical Implications

First, we traced the source of authoritarian leadership in supervisors to reveal the action mechanism of how authoritarian manager leadership affects unethical employee behavior. This is a new mechanism, charting the influence of senior managers on the behavior of ordinary employees in an organization, and that this could be caused by them influencing the leadership style supervisors and thereby influencing the behavior of employees. This also reveals how the leadership style of supervisors is formed. This article has shed more light on the psychological characteristics of supervisors, and how the leadership style of the senior managers may also have an impact on their leadership style.

Based on previous research, this study further proves that authoritarian leadership is a popular positive leadership technique among the three dimensions of paternalistic leadership (Liu et al., 2013), which can influence unethical employee behavior. This emphasizes that not only western leadership styles can affect unethical employee behavior (such as ethical leadership and responsibility leadership), but that Chinese authoritarian leadership can also have an impact on unethical employee behavior, thereby enriching the research on unethical employee behavior from the perspective of leadership.

The behavior of managers and employees does not occur in a vacuum, and the LMX and wider ethical climate provide an important organizational context that can have a trickle-down effect on employee behavior. LMX and the wider ethical climate are the lenses through which we interpret trickle-down behaviors, telling us under what circumstances this is more likely to occur and enabling us to identify possible ways in which unethical employee behavior can be reduced. This enriches research on the internal mechanisms in the behavior of senior managers and supervisors (including leadership style) and shows that LMX can also moderate the relationship between the two. Building upon previous studies, we have further demonstrated an internal mechanism of the influence of leadership style on employee behaviors, and the moderating effect of the situational factors of the organizational climate. In different organizational climates, leadership style has different effects on employee behaviors. More importantly, this study investigates the action mechanism between variables through the moderated mediation model, which can more comprehensively and systematically investigate the comprehensive action process of mediating variable and situational variables compared with a single study method, on mediating effect or moderating effect.

### Managerial Implications

The authoritarian leadership style should be used sparingly. Because authoritarian leadership has a trickle-down effect on the behavior of employees, it is, necessary to encourage leaders to authorize subordinates, communicate information with employees, change ways they criticize employees, and praise their abilities and contributions, in reducing unethical employee behavior. First, supervisors should not adopt an authoritarian leadership style because they are the direct leaders of employees and take direct responsibility for unethical employee behavior. Managers should also not adopt an authoritarian leadership style because authoritarian manager leadership has a profound impact on authoritarian supervisor leadership.

A highly ethical climate should be created in organizations. The ethical climate negatively moderates the relationship between authoritarian supervisor leadership and unethical employee behavior and moderates the mediating effect of authoritarian supervisor leadership. Therefore, as a team or organization, it is necessary to strive for a good ethical climate, fostering mutual care and concern, and reducing the motivations for engaging in unethical behavior. Employees should then be educated on how to abide by organizational rules and regulations, social laws, and professional codes to restrain unethical behaviors in employees.

### Limitations and Future Directions

In terms of research design, the data were collected immediately at the same time node, which may lead to common method deviation, even though the common method deviation was tested in this study. Data can be collected at several different time points in the future to better avoid common method deviation. The survey of authoritarian leadership may produce a social approbation effect; thus, in future research, we will use different methods (such as depth interviews and qualitative research methods, etc.) to collect data and to further explore the effects and action mechanisms of authoritarian leadership on unethical employee behavior.

The research conclusions of this paper show that authoritarian leadership brings negative effects and can lead to unethical employee behavior. However, some scholars have also concluded that authoritarian leadership can have positive effects. This suggests that authoritarian leadership does not always produce negative results, and in some cases, it may produce positive results. Therefore, we call on researchers to in future continue to explore the potential psychological mechanism and boundary conditions of mercy leadership that influence employee behavior and to examines how authoritarian leadership negatively affects employee behavior or the curve relationship between authoritarian leadership and employee behavior.

This paper studied the relationship between authoritarian leadership and unethical employee behavior in eastern organizations by using samples from China, which will affect the general applicability of our findings to other situations (Pellegrini and Scandura, 2008). However, we believe that this trickle-down effect of leadership style exists not only in authoritarian leadership but also in other leadership styles in Chinese society (benevolent leadership, moral leadership, etc.) and the leadership styles favored in the western world (transformational leadership, transactional leadership, etc.). We can therefore further expand the application and scope of this effect to, for example, abusive management and ethical leadership.

### Conclusion

Our study looked at the trickle-down effect of authoritarian leadership on unethical employee behavior, and the moderating effect of LMX and ethical climate. We find that the manager authoritarian leadership positively influences unethical behavior in employees, through the superior authoritarian leadership and LMX, and that this ethical climate moderates the relationship between them. Under high quality of LMX, the positive relationship between manager authoritarian leadership and supervisor authoritarian leadership is stronger, and when the ethical climate is high, the positive relationship between supervisor authoritarian leadership and employee unethical behavior is weaker.

## Data Availability Statement

The raw data supporting the conclusions of this article will be made available by the authors, without undue reservation.

## Ethics Statement

Ethical review and approval was not required for the study on human participants in accordance with the local legislation and institutional requirements. Written informed consent from the patients/participants or patients/participants legal guardian/next of kin was not required to participate in this study in accordance with the national legislation and the institutional requirements.

## Author Contributions

All authors listed have made a substantial, direct and intellectual contribution to the work, and approved it for publication.

### Conflict of Interest

The authors declare that the research was conducted in the absence of any commercial or financial relationships that could be construed as a potential conflict of interest.
